# Trismus Nascentium

**Published:** 1846-07

**Authors:** 


### TRISMUS NASCENTIUM.

Dr. Sims, of Montgomery, Alabama, has written an article on the *Lock-jaw of Infants*, which appeared in the American Journal of Medical Sciences, and has since been issued by him in a neat pamphlet of 20 pages. This affection, he holds, has its origin in displacement of the cranial bones, during labor, and keeping the child on its back, after birth—as he expresses it, in *bony displacement, and dorsal decubitus*." The veins of the cerebellum and spinal cord are thus pressed and ligatured. His remedy is a very simple one, and consists in providing "a nice soft pillow of feathers for each infant, and having it changed frequently from side to side, never allowing it to remain for any length of time upon its back." Let it be tried in the region where the disease is so fatal. Dr. Sims mentions a planter in Alabama, who lost five or six negro children from lock-jaw in as many months.

